# Physical Therapy Is an Underutilized Health Resource for Children with Cancer: A Retrospective Study Identifies Facilitators for Improvement

**DOI:** 10.3390/healthcare14010020

**Published:** 2025-12-21

**Authors:** Victoria Marchese, Lauren Savage, Kai Sun, Madhurika Situt, Teresa York, Rachel Reoli

**Affiliations:** 1 Department of Physical Therapy and Rehabilitation Sciences, School of Medicine, University of Maryland, Baltimore, MD 21201, USA; 2 Pediatric Hematology/Oncology Clinic, University of Maryland, Baltimore, MD 21201, USA

**Keywords:** physical therapy, pediatrics, oncology, utilization, rehabilitation, vincristine, ifosfamide

## Abstract

**Purpose:** Childhood cancers are the cause of treatment-associated morbidities, impairing functional mobility, participation and quality of life. Physical therapy is known to have a positive impact on health and well-being. Unfortunately, physical therapy is not utilized to its capacity. Thus, the aim of our study is to assess variables that facilitate utilization of physical therapy for children with oncological diagnoses across the continuum of care. **Methods:** A retrospective observational study of medical records was completed for children who received care at a large medical system. Descriptive statistics and multiple logistic regressions were performed. **Results:** Record review identified 16,975 episodes of care for 693 children. Of the 16,975, 240 included a referral to physical therapy. Of the 240, 178 used physical therapy. Presence of pain (odds ratio (OR) 20.026, *p* < 0.001), and being prescribed Ifosfamide or Daunorubicin (OR 28.213, *p* < 0.001; OR 15.439, *p* < 0.001, respectively) increased the likelihood of using physical therapy. **Conclusions:** We confirmed that physical therapy is underutilized for children with oncological diagnoses. However, clinical and socioeconomic variables were identified that facilitate use of physical therapy.

## 1. Introduction

Cancers are the leading cause of death by disease in children and adolescents both in the United States and globally, and are a primary source of treatment-associated morbidities [1,2]. Five-year survival rates have improved significantly for pediatric cancers in the last few decades from 63% in the 1970s to 83% in the 2000s [3]. The most common pediatric cancers are leukemia, central nervous system (CNS) tumors, lymphomas, and sarcomas [4,5,6]. Increases in survival rates are largely attributable to advancements in treatments including surgery, radiation, chemotherapy, targeted therapy and immunotherapy [7]. Despite the decrease in overall mortality, functional outcomes for childhood cancer differ depending on the type of malignancy, presence of metastases, age of clinical onset, acquisition of developmental milestones, anatomical site, stage of the disease, and presence or absence of somatic mutations [8]. Physical therapy has been shown to improve functional outcomes [9,10,11]; however, it is an underutilized resource [12] despite its cost benefit [13].

There is increasing evidence that factors in the environment where children live, play and grow may impact survival and quality of life for children with cancer [1,14,15]. Gilchrist et al. 2023 reported “…rehabilitation services are being increasingly provided to patients with ALL [Acute Lymphoblastic Leukemia] at a relatively low cost per patient, yet geographic variability in care utilization is evident” [13]. The area of deprivation index is a scale that ranks the conditions of neighborhoods, with a score of one reflecting the least disadvantaged groups and a score of ten representing the most disadvantaged [16,17]. Higher ADI is correlated with an increased risk of multimorbidity and indicates that neighborhood context contributes to health outcomes [18]. Furthermore, lack of health insurance or access to health services is associated with reduced care throughout the cancer treatment process including prevention, screening, and symptom management [19]. Yet it is known that access to healthcare increases survivorship [8].

While medical interventions result in higher survivorship across childhood cancer, the treatments can also lead to functional impairments. In addition to physical therapy, treatments for pediatric cancer patients include chemotherapy, radiation, targeted therapy, immunotherapy, and surgical removal, all of which have side effects that may impact mobility [20]. Long-term survivors of childhood cancers who experience persistent chronic fatigue have more depressive symptoms, anxiety, pain, and insomnia. They also engage in less physical activity and have an impaired ability to complete functional tasks [21,22]. Additionally, cardiovascular disease and neurologic complications often arise prematurely in pediatric cancer survivors because of the long-term effects of chemotherapy and radiation [23]. Specifically, chemotherapy is known to produce peripheral neuropathy, which can cause side effects such as numbness and weakness that impact functional mobility [24]. Approximately 83% of childhood ALL survivors experience neurological symptoms [25,26]. Common lasting side effects prevalent in survivors of central nervous system tumors include pain, seizures, and motor and sensory loss [27]. While loss of physical function is prevalent across all cancer types, musculoskeletal sarcoma often requires both pharmacological and surgical treatment [28]. These procedures may result in impairments in muscle properties including muscle strength and size, and neuromuscular activation. These functional deficits can limit a patient’s participation in school, work, and recreation [29,30]. It is known that physical therapy improves these multisystem impairments and increases functional mobility and quality of life for individuals with pediatric cancers [7,9,11,31,32,33,34,35]; thus, it is important to understand facilitators and barriers to physical therapy access and utilization.

Despite the broad range of functional impairments associated with oncology treatments, there is a lack of consistency in referring and providing rehabilitation services for childhood cancer survivors. Current guidelines suggest referral to physical therapy for comorbidity management [36], whereas others suggest a more frequent rehabilitation schedule for prevention [37]. It is well documented in the literature that physical therapy during and after treatment is feasible [33], effective [9,10], and can improve functional mobility for these individuals. Despite these findings, Rodwin et al. reported that only 27.2% of pediatric ALL patients overall and 58.9% of those with neuromuscular conditions received inpatient physical therapy within a year of first being admitted for cancer treatment [12]. The variables that impact referral and use of physical therapy services for pediatric oncology patients undergoing either active treatment or long-duration survivorship are largely unknown. Studies have found that factors including higher educational level, employment, and urban environment were correlated with a higher likelihood of using physical therapy [38]. However, pediatric patients with cancer face different challenges than the general population. Therefore, there is a critical gap in knowledge regarding the underlying factors affecting the referral and use of physical therapy among patients with childhood cancers. Knowledge of these variables could positively impact access to care. Thus, the aim of our study is to assess facilitators to utilization of physical therapy for children with oncological diagnoses across the continuum of care. Identification of these variables may foster precision-based rehabilitation to ultimately improve quality of life.

## 2. Materials and Methods

### Study Design

A retrospective observational study design was utilized. Following Institutional Review Board (IRB) approval, HP-00111699, electronic medical records were retrospectively accessed for children who received care for oncological diagnoses, from twenty medical center sites, within a single healthcare system, across the continuum of care (acute care to outpatient settings). Electronic medical records were retrieved in one timepoint by an investigator from the University’s Institute for Clinical and Translational Research using medical record variables defined by the primary investigator. Medical record variables included age, race, gender, height, weight, insurance, area of deprivation index, admission type, diagnosis (all codes for cancer), pain, physical therapy referral orders (including provider notes), physical therapy evaluation, medications (ifosfamide, daunorubicin, vincristine, doxorubicin, vinblastine, carboplatin, cisplatin, epirubicin, idarubicin, mitoxantrone, valrubicin). Ages were divided into five groups based on years of age (0–3, 4–6, 7–10, 11–15, 16–18). Area Deprivation Index (ADI) followed the traditional ADI numerical scale of one (least disadvantaged) to ten (most disadvantaged). All ADIs were included to better understand the population receiving medical care [17]. Once collected, electronic medical record data was stored and analyzed according to the University’s IRB protocol. The Strengthening the Reporting of Observational Studies in Epidemiology guidelines were followed. Participants were included if they had a diagnosis of pediatric cancer and received medical care from 1 January 2016 to 1 July 2024. Ages birth to eighteen were included. Per IRB protocol, the study was deemed exempt and consent waived.

Using the results of prior literature that identified variables associated with functional outcomes [39], it was identified that a sample size of 650 participants was required for a power of 0.80. Characteristic factors of subjects including age in groups, ADI in groups, insurance, different medication uses, pain, and admission (inpatient vs. outpatient), were reported as frequency. Chi-square tests were used for the comparisons. The multiple logistic regression models with random effects for individuals used to account for the non-independence of multiple episodes from the same patient (PROC GLIMMIX Procedure) were used to examine the possible impacts of characteristics of subjects to overall referral to physical therapy and overall physical evaluation, respectively. Effects were shown with odds ratios. Episodes of medical care were used for each model. Episodes of medical care included any admission to an inpatient or outpatient service; thus, for physical therapy, one referral would be necessary per episode of medical care. Given the multiple episodes of medical care for several patients, multiple logistic regression mixed models were used to address impacts of characteristics of subjects to referral and use of physical therapy to account for the non-independence of multiple episodes from the same patient. The clustering was addressed by including a random effect for individual. The variables included in the models are age, ADI, insurance, pain or not, inpatient location or not, Ifosamide use, Daunorubicin use, Vincristine use, and Doxorubicine use. Per the significant level and the clinical importance, the covariates were selected. The Hosmer–Lemeshow-type test was conducted to check the goodness of fit of the model. No missing data handling was conducted; missing data was automatically excluded from the analysis and did not substantially reduce sample size.

All analyses were performed using Statistical Analysis System (SAS) (v. 9.4, SAS Institute, Inc., Cary, NC, USA) with a significance level of 0.05.

## 3. Results

### 3.1. Descriptive Characteristics

Retrospective data analysis of medical records identified 16,975 episodes of medical care from 1 January 2016 and 31 March 2024, for 693 children with pediatric oncological diagnoses, which formed the denominators for episode-level analysis of physical therapy referral and use. Out of the 16,975 episodes of medical care, 240 episodes included a referral to physical therapy. Blood cancers accounted for 177 episodes; 15 were cancers of the nervous system, 32 were sarcomas, and 16 episodes were undefined or other. Of the 240 episodes that received referrals to physical therapy, 178 episodes used physical therapy services.

There was a significant difference in physical therapy referrals based on age (*p* = 0.05), ADI state rank (*p* < 0.001), insurance (*p* < 0.001), pain (*p* ≤ 0.001), medications (Vincristine (*p* < 0.001), Ifosfamide (*p* = 0.001), Daunorubicin (*p* < 0.001), Doxorubicin (*p* = 0.02), Idarubicin (*p* < 0.001)), and location (acute care versus outpatient) (*p* ≤ 0.001). Descriptive variables that were significant for children who used physical therapy services included ADI state rank (*p* < 0.001), insurance (*p* < 0.001), pain (*p* < 0.001), medications (Vincristine, Cisplatin, Ifosfamide, Daunorubicin, Doxorubicin, Idarubicin, and Mitoxantorone all (*p* < 0.001)) and location (acute care versus outpatient) (*p* ≤ 0.001). See Table 1 and Table 2.

### 3.2. Logistic Regression

Logistic regression found seven variables that were barriers to referral to physical therapy and four variables that facilitate d physical therapy. Barriers to physical therapy referral included being uninsured (OR 0.402, CI 0.198–0.814, *p* = 0.011), least disadvantaged ADI ranks (ADI 2 OR 0.159, CI 0.052–0.481, *p* = 0.001; ADI 3 OR 0.361, CI 0.143–0.915, *p* = 0.032; ADI 4 OR 0.240, CI 0.075–0.767, *p* = 0.016), and most disadvantaged ADI ranks (ADI 8 OR 0.363, CI 0.161–0.817, *p* = 0.014; ADI 9 OR 0.303, CI 0.121–0.755, *p* = 0.01; ADI 10 OR 0.309, CI 0.132–0.723, *p* = 0.007). Facilitators to physical therapy referral included presence of pain (OR 2.327, CI 1.592–3.401, *p* < 0.0001), being in the acute care setting (OR 1.792, CI 1.017–3.158, *p* = 0.044), and the medications Ifosfamide and Vincristine (OR 4.464, CI 1.433–13.9, *p* = 0.01; OR 1.69, CI 1.202–2.377, *p* = 0.003, respectively). See Table 3 for variables associated with referral of physical therapy.

Logistic regression identified three variables that impacted use of physical therapy: insurance, pain, and medication. Lack of insurance was a barrier to physical therapy use (uninsured, OR 0.06, CI 0.007–0.490, *p* = 0.009). Presence of pain increased physical therapy use (OR 20.026, CI 13.665–29.349, *p* ≤ 0.0001). Likewise, the medications Ifosfamide and Daunorubicin facilitated physical therapy use (OR 28.213, CI 12.031–66.161; OR 15.439, CI 5.454–43.705, respectively). See Table 4 for variables associated with use of physical therapy. The wide confidence intervals for Ifosomide and pain (as a predictor of physical therapy use) were due to very few occurrences of referral or use in those categories, as shown in Table 1 and Table 2. The Hosmer–Lemeshow-type test was conducted to check the goodness of fit of the model, with a *p*-value of 0.34, indicating a good fit.

## 4. Discussion

As survival rates improve, childhood cancers have become the leading cause of treatment-associated morbidities in the United States [1,2]. Children with oncological diagnoses experience impairments in multiple body systems, from both the cancer as well as from the life-saving medical interventions [20,21,22,23,24,25,26,27,28,29]. These impairments impact functional mobility, participation and quality of life [25]. Physical therapy is known to positively impact impairments and reduce secondary complications throughout the lifespan [7,9,11,30,31,32,33,34]. Thus, the aim of our study was to assess facilitators to access and use of physical therapy for children with cancer. We identified that for children with oncological diagnoses 240 of the 16,975 episodes of medical care had a referral to physical therapy. Additionally, of the 240 episodes that did receive a physical therapy referral, 178 episodes had associated physical therapy treatments. The American Physical Therapy (APTA) Health Promotion and Wellness Network states “the purpose…is to facilitate the profession’s role in transforming society and physical therapist practice by connecting people and knowledge to develop and disseminate best practices in prevention, health promotion, and wellness for all individuals and populations” [40]. In support of the APTA, our study identified both facilitators and barriers to physical therapy referrals and use for children with oncological diagnoses. Implementation of these variables into clinical settings may aid in the use of physical therapy services.

One of these variables, ADI, was found to be a barrier to referral to physical therapy. We identified ADI 5 to be the most common ADI served by the medical system. Interestingly, children housed in ADIs that were ranked as least disadvantaged (ADIs of two, three, and four) and children that were ranked as the most disadvantaged (ADIs eight, nine, and ten) had a lower likelihood of being referred to physical therapy services as compared to those with ADIs on the very least disadvantaged end (ADI one) or in the middle range (ADIs six and seven). As aforementioned, it is known that the general population with disadvantaged ADIs has reduced access to physical therapy [31]. Additionally, the results of our study collaborate with the findings by Gilchrist et al. that rehabilitation services are not equally provided across geographic regions for children with oncological diagnoses [13]. However, surprisingly, we found that children living in some of the least disadvantaged ADIs (ADI two, three and four) were also not likely to be referred to physical therapy services. Given this finding, we hypothesize that providers may assume children with lower ranking ADIs may already have access to home, community and school-based services other than physical therapy, such as a community recreational center or gymnasium, lowering the perceived need for physical therapy; however, our findings would lead to encouraging clinicians to communicate with patients regardless of ADI when physical therapy is indicated, and not assume that resources are or are not available.

A second variable, which may be related to ADI, that our study identified as significant to receiving physical therapy, is insurance. Being uninsured was a barrier to both referral and use of physical therapy. It is known that being uninsured or having gaps in insurance negatively impacts cancer care and survival rates [14]. Therefore, it is imperative that healthcare providers incorporate members of the multidisciplinary team, e.g., social work, to assist with opportunities for children and families to receive financial assistance for medical care, including physical therapy services. By sharing resources and opportunities for insurance coverage, patients may be more likely to be referred to and use physical therapy to remediate primary impairments and prevent secondary complications.

Unlike ADI and insurance, the presence of pain was a facilitator to referral and use of physical therapy. Our findings align with the APTA “Choose Physical Therapy” initiative which states “physical therapists are movement experts who treat pain and improve quality of life through hands-on care, patient education, and prescribed movement. They help people manage or eliminate pain and reduce the need for surgery and pain medications, such as opioids. By increasing physical activity you can reduce your risk of developing many chronic diseases” [40]. Our results are similar to other findings that support the use of PT as part of a multidisciplinary team for pain management in children with oncological diagnoses [41] and show a positive implementation of the APTA initiative with increased referral and use of physical therapy for children with pediatric oncology diagnoses and pain.

As children with pediatric cancers are living longer due to medical interventions and pharmacological treatments [3], short and long-term side effects, in addition to pain, may also impact functional mobility and quality of life. Thus, we examined the impact of medications on referral and use of physical therapy [42]. We found that being prescribed the medications Ifosfamide and Vincristine increased referrals to physical therapy and Ifosfamide and Daunorubicin facilitated use of physical therapy services. The increase in referrals and use of physical therapy with these medications is in agreement with the Children’s Oncology Group’s Survivorship Guidelines, which state “physical therapy referral for patients with symptomatic neuropathy… for non-pharmacologic pain management, range of motion, strengthening, stretching, functional mobility” [43]. Additionally, our findings align with Tanner et al.’s systematic review which reported that chemotherapy-induced peripheral neuropathy impacts physical function and quality of life for survivors of pediatric cancers and thus, supportive care interventions should be implemented [24]. Therefore, the results of our study support that when medications with mobility impacting side effects such as fatigue and/or neuropathy are being prescribed, physical therapy is being offered and used by children with oncological diagnoses.

While our study identified facilitators and barriers for referral and use of physical therapy, it is important to note several limitations. Despite the large sample size, data was retrieved from a single medical system located in one state. Thus, ADI information may vary across regions and medical systems and does not capture physical therapy use outside of the medical system. Additionally, we opted to group all cancer types together to obtain a complete picture of use of physical therapy for patients with pediatric oncological diagnoses. However, different diagnoses may have functional impairments, treatments, side effects, and outcomes specific to the pathology [7,8]. Additionally, a limiting factor could be electronic medical record misclassification and the lack of standardized functional outcome measures to directly capture physical therapy needs. Thus, future research would benefit from assessing multiple hospital systems across ADIs, as well as analyzing specific types of childhood cancers.

## 5. Conclusions

Our study identified that while physical therapy is underutilized for children with oncological diagnoses across the continuum of care; children who did receive physical therapy had facilitatory variables that aligned with other healthcare governing bodies and guidelines. Thus, future studies would benefit from implementation of these findings into the clinical setting to assess if referrals and use of physical therapy increase with knowledge of these variables.

## Figures and Tables

**Table 1:** Variables and Percentages of PT Referrals and Use.

Variable	Overall	Referral	Referral: *p*-Value	Use	Use: *p*-Value
**Age**			0.050 *		0.110
<3	2612	39 (1.493)		27 (1.034)	
4–6	3354	35 (1.043)		28 (0.845)	
7–10	3935	69 (1.753)		32 (0.813)	
11–15	4469	53 (1.186)		59 (1.320)	
16–18	2605	44 (1.689)		32 (1.228)	
**Gender**			0.720		0.110
Male	9317	129 (1.385)		87 (0.934)	
Female	7658	111 (1.449)		91 (1.188)	
**ADI Group**			<0.001 *		<0.001 *
1	926	34 (3.671)		7 (0.756)	
2	1405	7 (0.498)		10 (0.712)	
3	1255	16 (1.275)		8 (0.637)	
4	926	6 (0.648)		3 (0.324)	
5	1478	39 (2.639)		14 (0.947)	
6	1919	33 (1.719)		18 (0.938)	
7	1268	27 (2.129)		28 (2.208)	
8	2422	32 (1.321)		22 (0.908)	
9	1688	19 (1.256)		27 (1.599)	
10	2566	24 (0.935)		27 (1.052)	
**Insurance**			<0.001 *		<0.001 *
Medicaid	6763	121 (1.789)		116 (1.715)	
Private	4597	96 (2.088)		61 (1.327)	
Uninsured	5615	23 (0.409)		1 (0.018)	
**Pain**			<0.001 *		<0.001 *
Not present	6998	41 (0.586)		6 (0.086)	
Present	9977	199 (1.995)		172 (1.724)	
**Location**			<0.001 *		<0.001 *
Outpatient	6998	36 (0.514)		6 (0.086)	
Acute Care	9977	204 (2.045)		172 (1.724)	

Legend: Age (years); ADI: Area of Deprivation Index; () percentage; * significant at the level of a 0.05.

**Table 2:** Medication and Percentages of PT Referrals and Use.

Variable	Overall	Referral	Referral: *p*-Value	Use	Use: *p*-Value
**Vincristine**			<0.001 *		<0.001 *
No	15,086	177 (1.173)		140 (0.928)	
Yes	1889	63 (3.335)		38 (2.011)	
**Cisplatin**			0.610		<0.001 *
No	16,932	239 (1.412)		170 (1.004)	
Yes	43	1 (2.326)		8 (18.60)	
**Ifosfamide**			<0.001 *		<0.001 *
No	16,914	236 (1.395)		161 (0.952)	
Yes	61	4 (6.557)		17 (27.869)	
**Daunorubicin**			<0.001 *		<0.001 *
No	16,924	236 (1.394)		163 (0.963)	
Yes	51	4 (7.843)		15 (29.412)	
**Doxorubicin**			0.020 *		<0.001 *
No	16,626	230 (1.383)		165 (0.992)	
Yes	349	10 (2.865)		13 (3.725)	
**Idarubicin**			<0.001 *		<0.001 *
No	16,674	239 (1.433)		177 (1.061)	
Yes	1	1 (100)		1 (100)	
**Mitoxantrone**			0.811		<0.001 *
No	16,971	240 (1.414)		174 (1.025)	
Yes	4	0 (0)		4 (100)	

Legend: () percentage; * significant at the level of a 0.05.

**Table 3:** Multiple Logistic Regression Mixed Models: Variables Associated with Referral to Physical Therapy.

Variable	OR	CI Lower	CI Upper	*p*-Value
Gender male (female)	0.903	0.580	1.407	0.652
Insurance private (Medicaid)	0.882	0.565	1.379	0.583
Insurance uninsured (Medicaid)	0.402	0.198	0.814	0.011 *
ADI rank 1 (5)	0.668	0.247	1.809	0.427
ADI rank 2 (5)	0.159	0.052	0.481	0.001 *
ADI rank 3 (5)	0.361	0.143	0.915	0.032 *
ADI rank 4 (5)	0.240	0.075	0.767	0.016 *
ADI rank 6 (5)	0.556	0.251	1.234	0.149
ADI rank 7 (5)	0.514	0.208	1.271	0.150
ADI rank 8 (5)	0.363	0.161	0.817	0.014 *
ADI rank 9 (5)	0.303	0.121	0.755	0.010 *
ADI rank 10 (5)	0.309	0.132	0.723	0.007 *
Pain yes (no)	2.327	1.592	3.401	<0.0001 *
Inpatient yes (no)	1.792	1.017	3.158	0.044 *
Ifosfamide yes (no)	4.464	1.433	13.900	0.010 *
Daunorubicin yes (no)	1.330	0.420	4.206	0.628
Vincristine yes (no)	1.690	1.202	2.377	0.003 *
Doxorubicin yes (no)	1.161	0.579	2.330	0.673
Age 4–6 (0–3)	0.697	0.393	1.237	0.218
Age 7–10 (0–3)	0.728	0.403	1.314	0.292
Age 11–15 (0–3)	0.698	0.380	1.283	0.247
Age 16–18 (0–3)	1.222	0.637	2.344	0.546

ADI: area of deprivation index; OR: odds ratio; CI: confidence interval; () indicates reference group; age in years. * significant at the level of a 0.05.

**Table 4:** Multiple Logistic Regression Mixed Models: Variables Associated with Use of Physical Therapy.

Variable	OR	CI Lower	CI Upper	*p*-Value
Gender male (female)	1.090	0.605	1.964	0.773
Insurance private (Medicaid)	0.611	0.327	1.142	0.123
Insurance uninsured (Medicaid)	0.058	0.007	0.490	0.009 *
ADI rank 1 (5)	0.604	0.124	2.932	0.532
ADI rank 2 (5)	0.663	0.171	2.568	0.552
ADI rank 3 (5)	0.863	0.228	3.267	0.828
ADI rank 4 (5)	0.316	0.050	1.983	0.219
ADI rank 6 (5)	0.797	0.245	2.591	0.706
ADI rank 7 (5)	1.291	0.374	4.463	0.686
ADI rank 8 (5)	0.901	0.282	2.872	0.860
ADI rank 9 (5)	0.972	0.296	3.192	0.962
ADI rank 10 (5)	0.615	0.196	1.929	0.405
Pain yes (no)	20.026	13.665	29.349	<0.0001 *
Inpatient yes (no)	2.318	0.852	6.303	0.099
Ifosfamide yes (no)	28.213	12.031	66.161	<0.0001 *
Daunorubicin yes (no)	15.439	5.454	43.705	<0.0001 *
Vincristine yes (no)	0.976	0.576	1.655	0.929
Doxorubicin yes (no)	1.965	0.926	4.167	0.078
Age 4–6 (0–3)	1.274	0.582	2.792	0.545
Age 7–10 (0–3)	0.790	0.347	1.799	0.575
Age 11–15 (0–3)	0.736	0.323	1.676	0.466
Age 16–18 (0–3)	1.336	0.556	3.209	0.517

ADI: area of deprivation index; OR: odds ratio; CI: confidence interval; () indicates reference group; age in years. * significant at the level of a 0.05.

## Data Availability

The data sets presented in this article are not readily available given the nature of the data, protected patient health information.

## Abbreviations

The following abbreviations are used in this manuscript:

CNS	Central Nervous System
ALL	Acute Lymphoblastic Leukemia
ADI	Area of Deprivation Index
IRB	Institutional Review Board
OR	Odds Ratio
CI	Confidence Interval

## References

[1] Aristizabal P., Winestone L.E., Umaretiya P., Bona K. (2021). Disparities in Pediatric Oncology: The 21st Century Opportunity to Improve Outcomes for Children and Adolescents With Cancer. Am. Soc. Clin. Oncol. Educ. Book.

[2] Zahnreich S., Schmidberger H. (2021). Childhood Cancer: Occurrence, Treatment and Risk of Second Primary Malignancies. Cancers.

[3] Siegel D.A., Richardson L.C., Henley S.J., Wilson R.J., Dowling N.F., Weir H.K., Tai E.W., Lunsford N.B. (2020). Pediatric cancer mortality and survival in the United States, 2001–2016. Cancer.

[4] Whitehead T.P., Metayer C., Wiemels J.L., Singer A.W., Miller M.D. (2016). Childhood Leukemia and Primary Prevention. Curr. Probl. Pediatr. Adolesc. Health Care.

[5] Buhtoiarov I.N. (2017). Pediatric Lymphoma. Pediatr. Rev..

[6] Giannikopoulos P., Parham D.M. (2022). Pediatric Sarcomas: The Next Generation of Molecular Studies. Cancers.

[7] Marchese V., Thomas K.M., Morris G.S. (2017). Pediatric Oncology. Campbell’s Physical Therapy for Children.

[8] Erdmann F., Frederiksen L.E., Bonaventure A., Mader L., Hasle H., Robison L.L., Winther J.F. (2021). Childhood cancer: Survival, treatment modalities, late effects and improvements over time. Cancer Epidemiol..

[9] Linero-Bocanegra M., García-Conejo C., Ramírez-Pérez L., Cuesta-Vargas A.I., Trinidad-Fernández M. (2024). Effectiveness of Therapeutic Exercise for Children Undergoing Treatment for Cancer: A Systematic Review. Pediatr. Phys. Ther..

[10] Ospina P.A., Wiart L., Eisenstat D.D., McNeely M.L. (2020). Physical Rehabilitation Practices for Children and Adolescents with Cancer in Canada. Physiother. Can..

[11] Esbenshade A.J., Friedman D.L., Smith W.A., Jeha S., Pui C.-H., Robison L.L., Ness K.K. (2014). Feasibility and Initial Effectiveness of Home Exercise During Maintenance Therapy for Childhood Acute Lymphoblastic Leukemia. Pediatr. Phys. Ther..

[12] Rodwin R.L., Ma X., Ness K.K., Kadan-Lottick N.S., Wang R. (2022). Physical Therapy Utilization Among Hospitalized Patients With Pediatric Acute Lymphoblastic Leukemia. JCO Oncol. Pract..

[13] Gilchrist L., Tanner L., Finch M., Watson D., Hoover A., Turcotte L., Messinger Y. (2023). Utilization and Cost of Outpatient Rehabilitation Services for Pediatric Patients Treated for Acute Lymphoblastic Leukemia Using a Commercial Claims Database. Arch. Phys. Med. Rehabil..

[14] Chalfant V., Riveros C., Bradfield S.M., Stec A.A. (2023). Impact of social disparities on 10 year survival rates in paediatric cancers: A cohort study. Lancet Reg. Health Am..

[15] HTran Y., Coven S.L., Park S., Mendonca E.A. (2022). Social determinants of health and pediatric cancer survival: A systematic review. Pediatr. Blood Cancer.

[16] Kind A.J.H., Buckingham W.R. (2018). Making Neighborhood-Disadvantage Metrics Accessible—The Neighborhood Atlas. N. Engl. J. Med..

[17] University of Wisconsin School of Medicine and Public Health 2022 Area of Deprivation Index. https://www.neighborhoodatlas.medicine.wisc.edu/.

[18] Chamberlain A.M., Finney Rutten L.J., Wilson P.M., Fan C., Boyd C.M., Jacobson D.J., Rocca W.A., Sauver J.L.S. (2020). Neighborhood socioeconomic disadvantage is associated with multimorbidity in a geographically-defined community. BMC Public Health.

[19] Yabroff K.R., Reeder-Hayes K., Zhao J., Halpern M.T., Lopez A.M., Bernal-Mizrachi L., Collier A.B., Neuner J., Phillips J., Blackstock W. (2020). Health Insurance Coverage Disruptions and Cancer Care and Outcomes: Systematic Review of Published Research. JNCI J. Natl. Cancer Inst..

[20] Irestorm E., Schouten-van Meeteren A.Y.N., Van Gorp M., Twisk J.W.R., van Santen H.M., Partanen M., Grootenhuis M.A., van Litsenburg R.R.L. (2024). The development of fatigue after treatment for pediatric brain tumors does not differ between tumor locations. Pediatr. Blood Cancer.

[21] Zeller B., Loge J.H., Kanellopoulos A., Hamre H., Wyller V.B., Ruud E. (2014). Chronic Fatigue in Long-term Survivors of Childhood Lymphomas and Leukemia: Persistence and Associated Clinical Factors. J. Pediatr. Hematol. Oncol..

[22] Fantozzi P.M., Sprint G., Medina A.M. (2022). Pediatric sarcoma survivorship: A call for a developmental cascades approach. Dev. Psychopathol..

[23] Nayak L., Batchelor T.T. (2022). How I treat neurologic complications in patients with lymphoid cancer. Blood.

[24] Tanner L.R., Demers C., Schorr E.N., Rosenberg N.S., Hooke M.C., Turcotte L.M., Treat-Jacobson D. (2025). Impact of chemotherapy-induced peripheral neuropathy on physical function in pediatric cancer survivors: A systematic review. J. Cancer Surviv..

[25] Khan R.B., Hudson M.M., Ledet D.S., Morris E.B., Pui C.-H., Howard S.C., Krull K.R., Hinds P.S., Crom D., Browne E. (2014). Neurologic morbidity and quality of life in survivors of childhood acute lymphoblastic leukemia: A prospective cross-sectional study. J. Cancer Surviv..

[26] Turhan A.B., Tülin Fidan S., Yarar C., Nazlı Sakallı E., Özdemir Z.C., Bör Ö. (2018). Neurocognitive Consequences of Childhood Leukemia and Its Treatment. Indian J. Hematol. Blood Transfus..

[27] Pancaldi A., Pugliese M., Migliozzi C., Blom J., Cellini M., Iughetti L. (2023). Neuropsychological Outcomes of Children Treated for Brain Tumors. Children.

[28] Zhu Y., Wu X., Zhang W., Zhang H. (2023). Limb-salvage surgery versus extremity amputation for early-stage bone cancer in the extremities: A population-based study. Front. Surg..

[29] Qin E.S., Richards B., Smith S.R. (2023). Function in Cancer Patients: Disease and Clinical Determinants. Cancers.

[30] Rock K., Addison O., Gray V.L., Nelson C.M., Henshaw R.M., York T., Ruble K., Marchese V. (2023). Quantifying muscle strength, size, and neuromuscular activation in adolescent and young adult survivors of musculoskeletal sarcoma: Identifying correlates and responses to functional strengthening. Knee.

[31] Jerbi S.H.M., Alanazi S.N.S., Alanazi W.A.S., Mutlaq A.Y.H., Alotaibi F.S.M., Alenezi M.A.H. (2022). The Role of Physiotherapy in the Management of Lymphoma Patients: Systematic Review. Pharmacophore.

[32] Wacker K., Tanner L., Ovans J., Mason J., Gilchrist L. (2017). Improving Functional Mobility in Children and Adolescents Undergoing Treatment for Non–Central Nervous System Cancers: A Systematic Review. PM R.

[33] Gohar S.F., Comito M., Price J., Marchese V. (2011). Feasibility and parent satisfaction of a physical therapy intervention program for children with acute lymphoblastic leukemia in the first 6 months of medical treatment. Pediatr. Blood Cancer.

[34] Battanta N., Lange K., Kesting S.V., Marx-Berger D., Heesen P., Ober H., Onerup A., Pluijm S.M.F., Scheler E., Verwaaijen E.J. (2025). Supervised Physical Activity Interventions in Children and Adolescents with Cancer Undergoing Treatment—A Systematic Review. Curr. Oncol..

[35] Marchese V., Rock K., York T., Ruble K., Gray V.L. (2022). The Efficacy of Targeted Exercise on Gross Motor and Neuromuscular Performance in Survivors of Childhood Leukemia: A Pilot Study. Front. Pediatr..

[36] L’Hotta A.J., Randolph S.B., Reader B., Lipsey K., King A.A. (2023). Clinical practice guideline and expert consensus recommendations for rehabilitation among children with cancer: A systematic review. CA Cancer J. Clin..

[37] Myers A., Lenker H., Reoli R. (2024). Optimum Frequency for Physical Therapy Intervention in Pediatric Patients Undergoing Bone Marrow Transplantation in Acute Care Settings: A Systematic Review. Rehabil. Oncol..

[38] Braaten A.D., Hanebuth C., McPherson H., Smallwood D., Kaplan S., Basirico D., Clewley D., Rethorn Z. (2021). Social determinants of health are associated with physical therapy use: A systematic review. Br. J. Sports Med..

[39] Kenney M.O., Wilson S., Shah N., Bortsov A., Smith W.R., Little J., Lanzkron S., Kanter J., Padrino S., Owusu-Ansah A. (2024). Biopsychosocial Factors Associated with Pain and Pain-Related Outcomes in Adults and Children With Sickle Cell Disease: A Multivariable Analysis of the GRNDaD Multicenter Registry. J. Pain.

[40] (2024). APTA Health Promotion and Wellness Network. https://www.apta.org/apta-and-you/leadership-and-governance/member-engagement-groups/networks/network-prevention-health-promotion-wellness.

[41] Choose PT. https://www.choosept.com/why-physical-therapy/safe-pain-management.

[42] Snaman J.M., Baker J.N., Ehrentraut J.H., Anghelescu D.L. (2016). Pediatric Oncology: Managing Pain at the End of Life. Pediatr. Drugs.

[43] (2023). Long-Term Follow-Up Guidelines for Survivors of Childhood, Adolescent, and Young Adult Cancers. http://survivorshipguidelines.org/pdf/2023/COG_LTFU_Guidelines_Only_v6.pdf.

